# Correlation between remnant cholesterol and hyperuricemia in patients with type 2 diabetes mellitus: a cross-sectional study

**DOI:** 10.1186/s12944-024-02148-3

**Published:** 2024-05-25

**Authors:** Hainiao Lin, Jing Xu, Chenhuai Teng

**Affiliations:** 1 Department of Emergency Medicine, The Second Affiliated Hospital and Yuying Children’s Hospital of Wenzhou Medical University, Wenzhou, Zhejiang Province China; 2 Department of Endocrine, The Second Affiliated Hospital and Yuying Children’s Hospital of Wenzhou Medical University, Wenzhou, China

**Keywords:** Metabolic syndrome, Remnant cholesterol, Hyperuricemia, Diabetes mellitus, Uric acid

## Abstract

**Background:**

Remnant cholesterol (RC) has been known as an important factor for the assessment of the metabolic syndrome (Mets) risk. However, the correlation between RC and hyperuricemia (HUA) in type 2 diabetes mellitus (T2DM) remains unclear. This study aims to explore the correlation between RC and HUA in patients with T2DM.

**Methods:**

A total of 2956 patients with T2DM admitted to the Second Affiliated Hospital and Yuying Children’s Hospital of Wenzhou Medical University from 2020 to 2022 were included. The correlation between RC and HUA was evaluated with Spearman’s correlation, multiple logistic regression, subgroup analyses, receiver operating characteristic (ROC) curves analyses and generalized smooth curve fitting. Total cholesterol (TC) < 5.18mmol/L was defined as normal TC.

**Results:**

RC was correlated with uric acid in patients with T2DM (Spearman’s correlation coefficient = 0.279, *P* < 0.001). According to the multiple logistic regression analyses, there was an independent positive correlation between RC and HUA (OR = 1.63, 95%CI = 1.40, 1.90). In addition, a non-linear correlation between RC and HUA was identified. The area under the ROC curve (AUC) of RC (0.658, 95%CI = 0.635, 0.681) was the largest compared with those of low-density lipoprotein cholesterol (LDL-C), triglyceride (TG), high-density lipoprotein cholesterol (HDL-C) and TC. Subgroup analyses showed a more significant positive correlation among females or normal TC groups.

**Conclusion:**

Elevated RC is correlated with HUA in patients with T2DM significantly and positively. RC is better in its predictability for HUA than that of conventional lipid indexes.

**Supplementary Information:**

The online version contains supplementary material available at 10.1186/s12944-024-02148-3.

## Introduction

HUA is a metabolic abnormality syndrome caused by disturbance of purine metabolism [1]. Previous studies have indicated that HUA is closely associated with an increased risk of metabolic syndrome, cardiac death, chronic kidney disease (CKD), cardiovascular disease (CVD) and all-cause mortality [2, 3]. Currently, epidemiological investigations have revealed that the overall prevalence of HUA in China is 13.3% [4], with a notably higher occurrence in patients with diabetes mellitus (DM) [5]. The substantial rise in the prevalence of HUA poses a significant challenge to public health, with a considerable socioeconomic burden [6]. Therefore, the identification of risk factors associated with high uric acid levels in patients with DM and the discovery of potential valuable indexes can significantly enhance the management and treatment of chronic diseases.

Remnant cholesterol (RC), an innovative atherogenic lipoprotein, refers to the cholesterol content presenting in triglyceride-rich lipoproteins, predominantly comprising very low-density lipoproteins, chylomicron remnants, and intermediate-density lipoproteins. Typically, RC is determined by subtracting the levels of LDL-C and HDL-C from TC, as calculated from a standard lipid profile [7]. Notably, mechanistic evidence indicated that elevated concentrations of RC are associated with low-grade inflammation, which are genetically affected by insulin resistance (IR) [8–11]. A study of a subject on the epidemiology demonstrated that as the level of RC increases, there is a corresponding increase in the prevalence of T2DM, hypertension, and hypertriglyceridemia [12–15]. Furthermore, the correlation between RC and MetS is characterized by a positive feedback loop involving IR, chronic inflammation, hypertension and abnormal lipid metabolism [16–19]. RC has the impact on these factors and its reciprocal correlation with the results in the accelerated progression of MetS [16, 17].

In previous studies, the correlation between conventional lipid parameters such as TC or TG and HUA has been explored [20–23]. In addition, Wang et al. found a positive correlation between elevated RC and HUA in American adults [24]. However, the precise correlation between RC and HUA in patients with T2DM remains unclear. Consequently, this study aims to investigate the potential link between RC and HUA in patients with T2DM through a cross-sectional analysis, so as to determine the viability of RC as a novel and practical biomarker for the diagnosis of HUA.

## Methods

### Subjects and research design

In this cross-sectional study, a total of 2956 patients with T2DM admitted to the Department of Endocrinology of the Second Affiliated Hospital and Yuying Children’s Hospital of Wenzhou Medical University between January 2020 and August 2022 were included. The study was approved by the hospital’s Ethical Review Committee (Approval No.: LCKY2020-01), with written consent from all patients with T2DM.

In this study, the inclusion criteria consisted of a diagnosis for T2DM on the criteria established by the World Health Organization, a minimum age of 20 years, complete biochemical parameters, and clinical information. Exclusion criteria were as follows: (1) A history of using diuretics or other medications potentially impacting uric acid metabolism over the past two months; (2) Acute inflammatory or infection disease; (3) Acute diabetic complications such as ketoacidosis or hyperosmolar state (coma); (4) Chronic kidney disease accompanied by estimated glomerular filtration rate (eGFR) less than 60 mL/min; (5) Severe chronic illness, such as cardiovascular diseases and cancer.

### Biochemical and anthropometric measurements

Duration of diabetes (DD), history of hypertension, hypoglycemic drugs, lipid-lowering drugs (LLDs), alcohol intake, smoking habits and physical measurements, including waist circumference height, weight and blood pressure were collected at admission. Specifically, the definitions of alcohol status, hypertension, smoking and BMI were described in previous studies [25].

To obtain blood samples, 4–5 mL venous blood was collected on the following morning after patients fasted overnight for 12 h. LDL-C, serum uric acid, alanine aminotransferase (ALT), TC, glycosylated hemoglobin (HbA1c), TG, HDL-C, aspartate aminotransaminase (AST), albumin, creatinine, gamma-glutamyl transpeptidase (GGT) and fasting plasma glucose (FPG) were determined as previously described [12]. Blood lipids were measured with enzymatic method and Olympus automatic biochemical analyzer.

RC was calculated through the following formula: RC = TC—HDL-C—LDL-C. RC values were divided into four groups based on quartiles (Q1–Q4). HUA was defined as a uric acid level exceeding 420µmol/L in males and 360µmol/L in females [4]. The classification of HDL-C, TC, LDL-C and TG was determined in accordance with the Guidelines on the treatment and prevention of blood lipid abnormalities in Chinese adults [26]. TG at the cutoff value of 1.70 mmol/L was defined as normal TG and hypertriglyceridemia. TC at the cutoff value of 5.18 mmol/L was defined as normal TC and hypercholesterolemia. HDL-C at the cutoff value of 1.04 mmol/L was defined as normal HDL-C and low HDL-C. LDL-C at the cutoff value of 3.37 mmol/L was defined as normal LDL-C and high LDL-C.

### Statistical analysis

In this study, continuous data were expressed as weighted mean ± SD, while categorical variables were expressed as percentage. The patients were divided into four groups or quartiles based on the levels of RC. In order to evaluate the differences between each group, χ2 test was adopted for categorical variables, t-test or Mann-Whitney U test for continuous variables. The correlation between RC and the presence of HUA was assessed with Binary logistic regression models. In Model 1, there was no adjustment. In Model 2, there were adjustment for gender and age. Based on Model 2, BMI, waist circumference, SBP, DBP, HbA1c, ALT, GGT, serum creatinine, albumin, drinking, smoking, DD, LLDs, hypoglycemic drugs were added to Model 3 as covariates. Mediation analysis was performed on the parallel mediation model, with individual indicators serving as mediators. The potential impacts of gender, BMI, hypertension, age, TC, TG, HDL-C as well as LDL-C on the correlation between RC and HUA were examined through subgroup analyses. To investigate potential nonlinear correlations between RC and HUA probabilities, a smooth curve fitting approach was employed. The diagnostic accuracy of RC in detecting HUA was assessed through ROC analyses. Additionally, a sensitivity analysis was conducted to avoid the potential influence of LLDs on the correlation between RC and HUA, with a subgroup of patients without LLDs (*n* = 2185) being analyzed. EmpowerStats software and R were adopted for the statistical analysis, with the significance determination (*P* < 0.05).

## Results

### Baseline characteristics

A total of 2956 patients aged from 25 to 90 years old were included in this study, with the prevalence of HUA of 27.8%. The population characteristics of the patients based on serum RC quartiles (Q1: <0.34; Q2: 0.34–0.53; Q3: 0.53–0.80; Q4: >0.80) are presented in Table 1. Compared with the bottom quartile, the prevalence of HUA and hypertension was higher in those in the top quartile of RC, with elevated levels of body weight, waist circumference, systolic and diastolic blood pressure, FPG, creatinine, uric acid, TC, and TG. In contrast, the level of HDL-C were lower (*P* < 0.001) (Table 1).

**Table 1:** Characteristics of the study population based on RC quartiles

Characteristic	Q1	Q2	Q3	Q4	*P* value
Number	745	750	712	749	
Age, year	56.2 ± 16.0	60.2 ± 15.1	61.5 ± 14.2	57.6 ± 14.8	0.086
Duration of diabetes, year	5.5 ± 7.0	6.1 ± 7.2	7.7 ± 7.2	7.5 ± 8.2	< 0.001
Male, %	61.7	57.7	57.4	58.1	0.294
Height, cm	164.1 ± 10.0	163.5 ± 10.2	163.3 ± 10.3	164.2 ± 9.6	0.714
Weight, cm	63.6 ± 16.7	63.6 ± 15.9	64.7 ± 15.1	66.7 ± 15.7	< 0.001
Body mass index, Kg/m2	24.5 ± 22.1	24.0 ± 9.7	24.3 ± 8.2	24.7 ± 6.5	0.761
Waist circumference, cm	85.2 ± 11.0	86.5 ± 10.8	88.7 ± 30.1	88.3 ± 10.4	< 0.001
Systolic blood pressure, mmHg	138.5 ± 26.8	141.0 ± 26.7	141.2 ± 26.9	144.0 ± 26.3	< 0.001
Diastolic blood pressure, mmHg	82.6 ± 8.2	82.3 ± 8.4	84.0 ± 32.7	84.5 ± 8.0	< 0.001
Hypertension, %	39.2	47.0	52.6	55.1	< 0.001
Current smoking, %	34.3	29.4	25.4	24.5	0.260
Current drinking, %	24.9	22.9	22.8	17.6	0.586
Lipid-lowering drugs, %	18.6	21.5	35.4	29.5	< 0.001
Metformin, %	45.1	42.9	52.2	52.7	0.229
Insulin, %	29.4	20.8	37.8	35.8	0.169
Hyperuricemia, %	17.6	20.5	30.3	42.7	< 0.001
Hemoglobin A1c, mmol/L	9.4 ± 2.4	9.0 ± 2.2	8.8 ± 2.2	9.5 ± 2.3	0.690
FPG, mmol/L	6.71 ± 2.11	6.59 ± 1.86	6.81 ± 2.12	6.98 ± 1.98	0.018
ALT, U/L	29.3 ± 37.8	26.4 ± 27.7	26.5 ± 35.7	29.2 ± 32.2	0.936
AST, U/L	25.2 ± 21.1	25.2 ± 19.0	25.7 ± 27.3	26.7 ± 22.6	0.175
GGT, U/L	49.8 ± 136.8	41.2 ± 92.6	42.7 ± 93.9	55.7 ± 73.7	0.296
Albumin, g/dl	39.8 ± 4.1	39.8 ± 4.1	40.2 ± 5.2	40.2 ± 4.6	0.066
Creatinine, umol/L	66.1 ± 29.2	73.1 ± 50.7	80.5 ± 64.7	86.2 ± 71.5	< 0.001
Uric acid, umol/L	318.9 ± 103.3	329.5 ± 101.5	353.9 ± 109.8	387.1 ± 115.4	< 0.001
TC, mmol/L	4.39 ± 1.08	4.07 ± 1.10	4.21 ± 1.13	5.18 ± 1.48	< 0.001
TG, mmol/L	1.14 ± 0.47	1.30 ± 0.49	1.71 ± 0.66	3.64 ± 2.68	< 0.001
HDL-C, mmol/L	1.10 ± 0.34	1.07 ± 0.33	1.02 ± 0.30	0.93 ± 0.27	< 0.001
LDL-C, mmol/L	3.08 ± 1.02	2.57 ± 1.02	2.54 ± 1.03	2.69 ± 1.08	< 0.001

Values are mean±SD or number (%). *P* < 0.05 was deemed significant. BMI, body mass index; LLD, lipid-lowering drugs; FPG, fasting blood glucose; HbA1c, glycosylated hemoglobin; TC, total cholesterol; TG, triglyceride; HDL-C, High density lipoprotein cholesterol; LDL-C, Low density lipoprotein cholesterol; GGT, glutamyl transpeptidase

### Correlation between RC and metabolic parameters

**Fig. 1:**
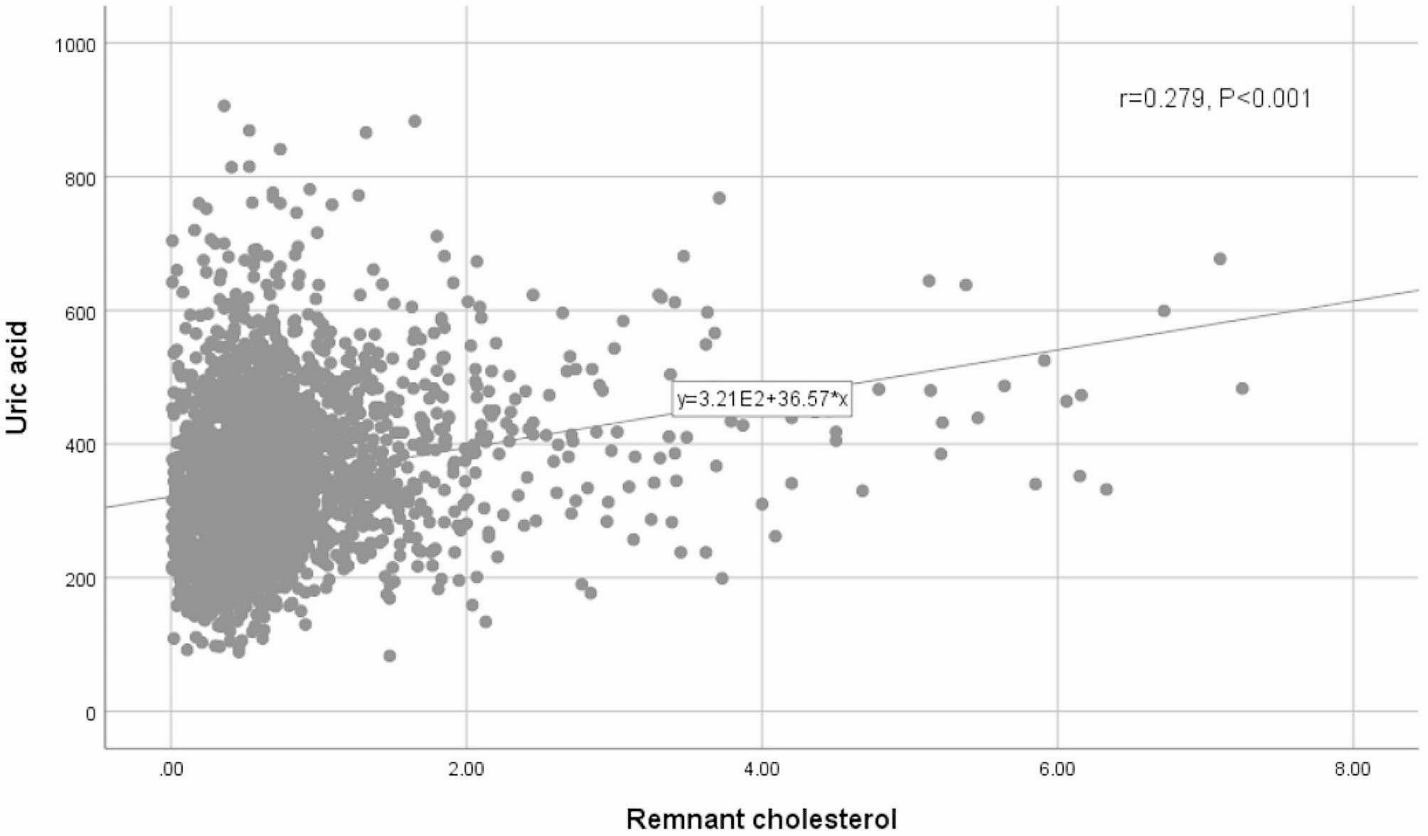
Scatter diagrams showing the correlation between the RC and uric acid

The correlation between RC and metabolic parameters measured by Spearman’s correlation coefficient, can be found in Table 2. It is evident that RC was positively correlated with BMI (*r* = 0.254, *P* < 0.001), WC (*r* = 0.209, *P* < 0.001), SBP (*r* = 0.077, *P* = 0.047), DBP (*r* = 0.149, *P* < 0.001), FPG (*r* = 0.111, *P* < 0.001), TC (*r* = 0.198, *P* < 0.001), TG (*r* = 0.688, *P* < 0.001), uric acid (*r* = 0.279, *P* < 0.001), and negatively correlated with HDL-C (*r*=-0.215, *P* < 0.001), LDL-C (*r*=-0.162, *P* < 0.001) (Fig. 1; Table 2).

**Table 2:** Spearmen’s correlation of RC levels with clinical and biochemical parameters

Variable	RC	
	*r*	*P*
BMI	0.254	< 0.001
WC	0.209	< 0.001
SBP	0.077	0.047
DBP	0.149	< 0.001
FPG	0.111	< 0.001
TC	0.198	< 0.001
TG	0.688	< 0.001
HDL-C	-0.215	< 0.001
LDL-C	-0.162	< 0.001
Uric acid	0.279	< 0.001

### Correlation between RC and HUA risk

Three logistic multivariate regression models were developed to examine the correlation between HUA and RC (Table 3 and Table S1). In the unadjusted model, RC was positively correlated with HUA probabilities [OR = 1.92, 95% CI: (1.69, 2.17)]. There was still the correlation in the Model 2 [OR = 1.92, 95% CI: (1.69, 2.17)] and Model 3 [OR = 1.63, 95% CI: (1.40, 1.90)]. Moreover, compared with the lowest level of RC (Q1) in Model 3 (P for trend < 0.001), HUA risk of the patients in quartiles 3 and 4 increased by 0.71 and 1.36, respectively.

**Table 3:** Association between RC and hyperuricemia in logistic regression analysis

	Model1 OR (95% CI) *P* value	Model2 OR (95% CI) *P* value	Model3 OR (95% CI) *P* value
RC	1.92 (1.69, 2.17), < 0.001	1.92 (1.69, 2.17), < 0.001	1.65 (1.42, 1.92), < 0.001
RC(Quartile)			
Q1	Reference	Reference	Reference
Q2	1.21 (0.94, 1.57), 0.147	1.22 (0.94, 1.58), 0.132	0.93 (0.64, 1.33), 0.926
Q3	2.04 (1.59, 2.61), < 0.001	2.07 (1.61, 2.65), < 0.001	1.71 (1.22, 2.39), 0.002
Q4	3.50 (2.76, 4.44), < 0.001	3.50 (2.76, 4.45), < 0.001	2.36 (1.71, 3.26), < 0.001
P for trend	< 0.001	< 0.001	< 0.001

Model I: None covariates were adjusted; Model II: gender and age were adjusted; Model III: BMI, waist circumference, SBP, DBP, HbA1c, ALT, GGT, serum creatinine, albumin, drinking, smoking, DD, LLDs, hypoglycemic drugs

### Subgroup analysis to assess the correlation between RC and HUA

**Fig. 2:**
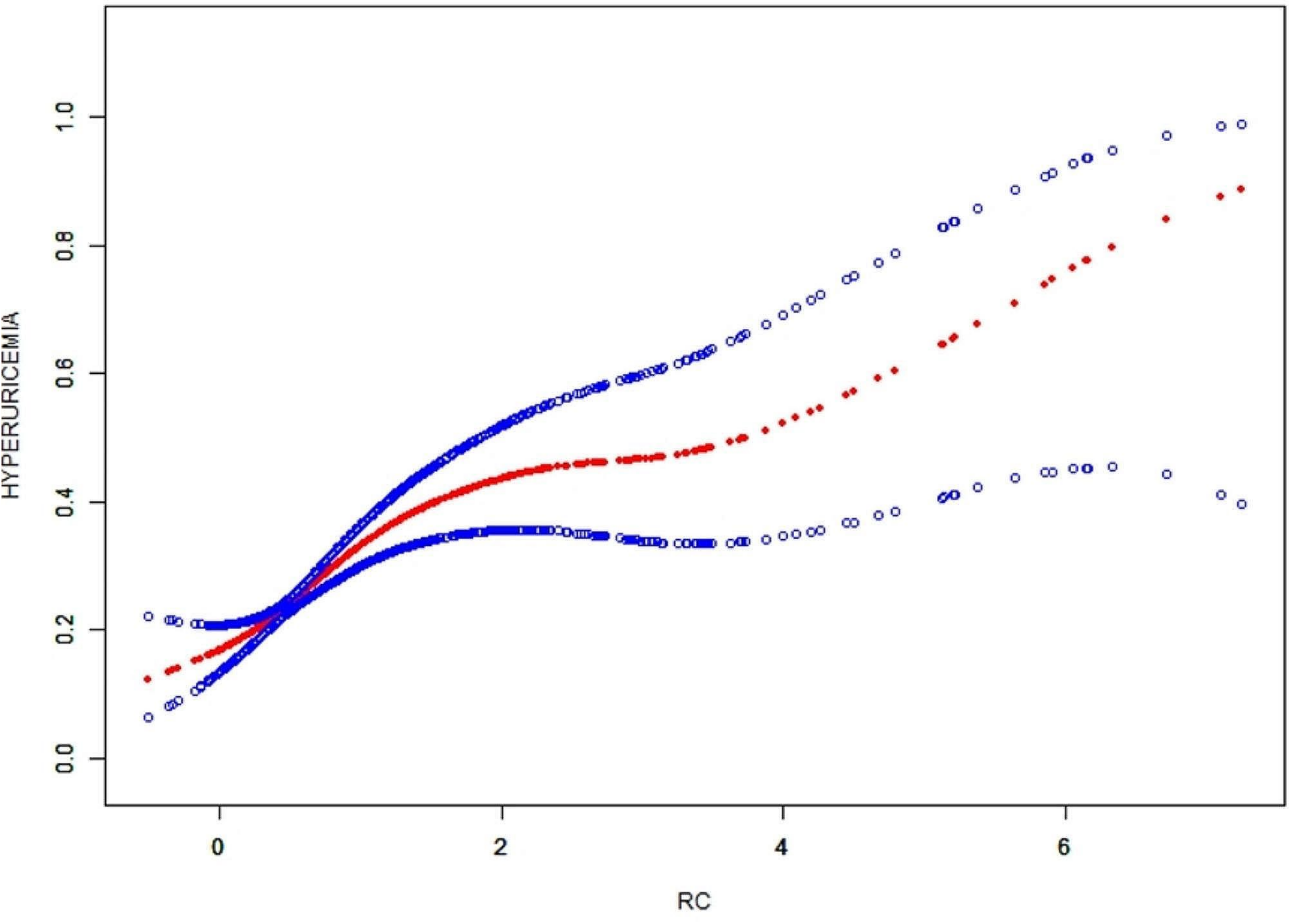
The smooth curve fit for the association between RC and hyperuricemia. Solid redline represents the smooth curve fit between variables. Blue bands represent the 95% of confidence interval from the fit

**Fig. 3:**
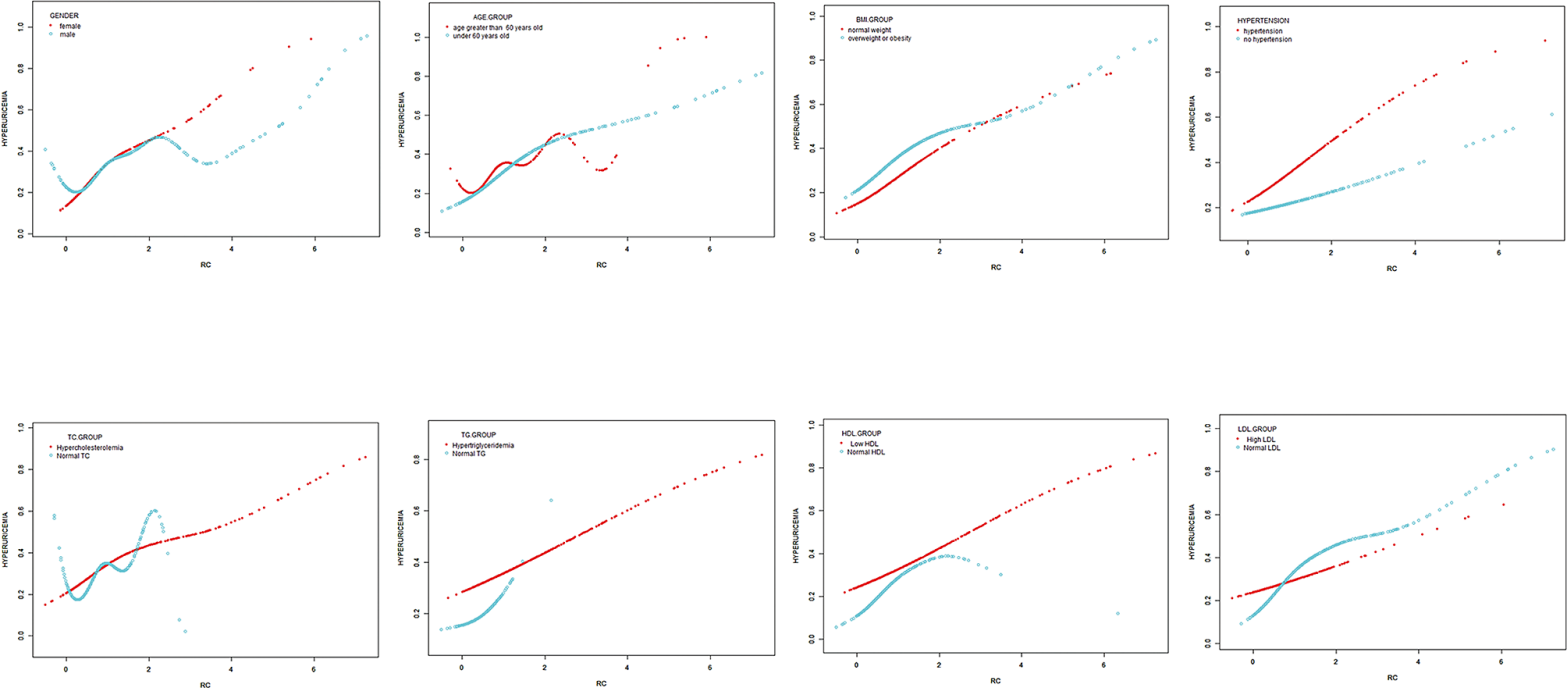
Subgroups analysis for the association between RC and hyperuricemia by gender, age, BMI, hypertension, TC, TG, HDL-C and LDL-C

A comprehensive subgroup analysis was performed to evaluate the consistency of the correlation between RC and HUA risk in different demographic contexts. As shown in Table 4, the correlation between RC and HUA risk was stronger among females and normal TC patients than that in males and hypercholesterolemia patients (*P* interaction < 0.05). In all patients, a positive correlation was found in the non-linear correlation, with inflection points of 7.443 (Fig. 2). In addition, Fig. 3 displays the smooth curves showing the positive correlation between RC and HUA in most groups.

**Table 4:** Association between RC and hyperuricemia stratified by gender, age, BMI, hypertension, TC, TG, HDL-C and LDL-C

	OR (95%CI) *p* value	*P* for interaction
Stratified by gender		0.041
Male	1.57 (1.31, 1.88), < 0.001	
Female	1.77 (1.32, 2.37), < 0.001	
Stratified by age		0.990
Age ≤ 60 years old	1.60 (1.32, 1.93), < 0.001	
Age > 60 years old	1.69 (1.29, 2.22), < 0.001	
Stratified by BMI		0.441
BMI < 24 kg/m^2^	1.80 (1.37, 2.38), < 0.001	
BMI ≥ 24 kg/m^2^	1.50 (1.24, 1.81), < 0.001	
Stratified by hypertension		0.051
No hypertension	1.39 (1.14, 1.72), 0.022	
Hypertension	2.00 (1.49, 2.70), < 0.001	
Stratified by TG		0.205
Normal TG	2.46 (1.23, 4.94), 0.011	
Hypertriglyceridemia	1.32 (1.14, 1.53), < 0.001	
Stratified by TC		0.041
Normal TC	2.10 (1.57, 2.83), < 0.001	
Hypercholesterolemia	1.50 (1.27, 1.77), < 0.001	
Stratified by HDL-C		0.138
Normal HDL-C	1.56 (1.12, 2.17), 0.009	
Low HDL-C	1.59 (1.36, 1.86), < 0.001	
Stratified by LDL-C		0.106
Normal LDL-C	1.80 (1.52, 2.12), < 0.001	
High LDL-C	1.41 (1.05, 1.90), 0.023	

Gender, age, BMI, hypertension, TC, TG, HDL-C, LDL-C (not adjusted for in the subgroup analyses), waist circumference, HbA1c, ALT, GGT, serum creatinine, albumin, drinking, smoking, DD, LLDs, hypoglycemic drugs were adjusted

### ROC analysis

**Fig. 4:**
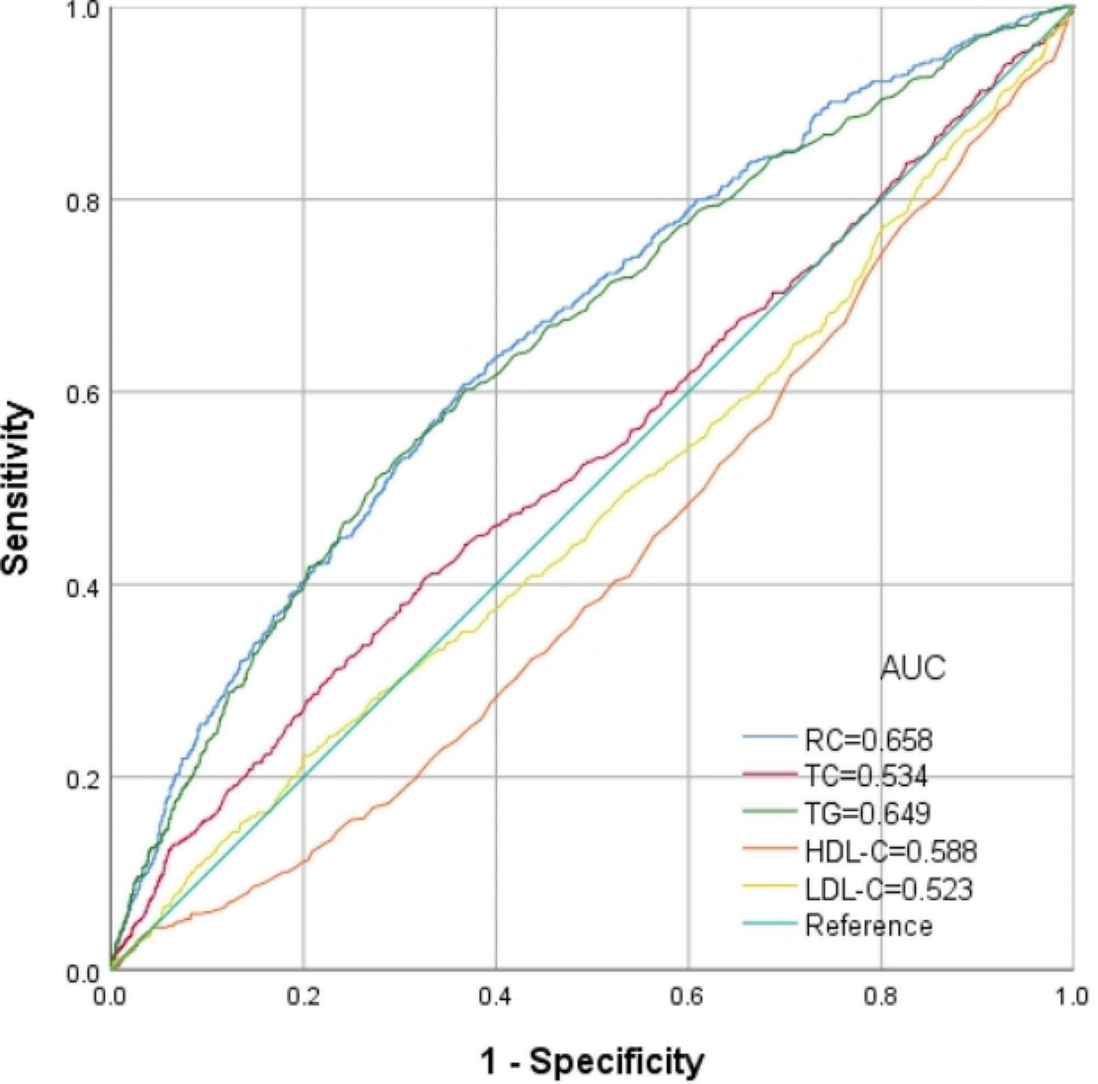
ROC analysis of RC to identify hyperuricemia risk

Figure 4 shows the ability of ROC of RC, HDL-C, TC, TC, LDL-C and TG in identifying HUA risk. The AUC for RC was significantly greater than TC, TG, HDL-C and LDL-C through the ROC analysis (0.658, 95%CI = 0.635, 0.681), with the sensitivity of 60.8%, the specificity of 63.3% and the cutoff of 0.54 (Table 5).

**Table 5:** The results of ROC analysis of RC, TC, TG, HDL-C and LDL-C for the diagnosis of hyperuricemia

Nutritional indices	Cut-off	Sensitivity (%)	Specificity (%)	AUC	95% CI
RC	0.54	60.8	63.3	0.658	0.635–0.681
TC	5.17	31.0	77.1	0.534	0.510–0.558
TG	1.66	59.6	63.7	0.649	0.625–0.673
HDL-C	0.98	53.9	60.7	0.588	0.565–0.610
LDL-C	2.19	68.6	38.3	0.523	0.507–0.544

### Sensitivity analysis

Considering the probable effect of LLDs on the correlation between RC and HUA, a sensitivity analysis was performed to determine this correlation after excluding the patients who took the LLDs (*n* = 771). In the multivariable-adjusted logistic model, RC was positively correlated with HUA probabilities (Supplementary Table 2).

## Discussion

The extensive studies have revealed a positive correlation between RC and an increased uric acid, and HUA risk among patients with T2DM. Moreover, the results of subgroup analyses indicated a robust positive correlation, particularly in females and patients with normal TC levels. Furthermore, the findings revealed a non-linear correlation between RC and HUA risk. In addition, RC has the superior predictive ability for HUA compared with conventional lipid parameters.

In view of the rising prevalence and substantial impact on various clinical disorders, HUA has become a significant public health issue [2, 27–29]. Cao et al. conducted a large-scale prospective cohort study involving 58,542 Chinese individuals. The study revealed an incidence of HUA of 12.1% with a median follow-up for 2.5 years. In a separate prospective cohort study conducted in China, Zhang et al. found an occurrence of HUA in 25.9% of participants over 6 years [30]. Notably, the community atherosclerosis risk study included 9451 Americans who often ate high fructose corn syrup, such as sugared soda water. It was reported that during the 6-year follow-up, the incidence rate of HUA was 34.8% [31]. This study on patients with T2DM (58.9 ± 15.2 years, 58.8% males), it observed 27.4% of T2DM adults with HUA. As a result of the significant shift towards Western dietary habits among the Chinese due to rapid lifestyle westernization, the prevalence of hyperuricemia is expected to rise in China, potentially resulting in severe health consequences. Therefore, investigating the risk factors associated with hyperuricemia is crucial for the early prevention and treatment of cardiovascular diseases.

The impact of dyslipidemia on the development of HUA has been investigated in various clinical and epidemiological studies. NHANES III indicated a significant correlation between TG and TC levels and UA levels in the serum of ordinary adults [22]. A retrospective population-based study involving 3884 medical examined patients collected from Gansu, China, revealed a positive correlation between elevated TG and HUA [21]. Recent studies has shown that abundant RC in triglyceride (TG) lipoprotein, such as intermediate-density lipoprotein, chylomicron remnants, and very-low-density lipoprotein [32], can contribute to various atherosclerotic effects, including the upregulation of proinflammatory cytokines, activation of monocytes, and increased production of thrombogenic factors [11, 32]. Adverse cardiovascular events associated with RC have been documented in numerous clinical studies. However, RC has been proposed as a potential means of identifying individuals at higher risk for T2DM, cardiovascular diseases, chronic kidney disease, fatty liver and Mets [33–38], no studies have reported on the correlation between the prevalence of HUA and the increased RC yet.

According to this study, RC was positively correlated with TG, DBP, BMI, FPG, WC, SBP and negatively correlated with HDL-C, which is consistent with previous studies. Additionally, it was found that the correlation between RC and TG was the strongest compared to that with RC and that with other components of MetS. This finding is consistent with previous studies, suggesting that TG is primarily transported by remnants and that the concentration of TG significantly increases with elevated levels of RC [14, 39]. In addition, it was observed that as RC levels increased, HDL-C levels decreased due to the exchange of triglycerides and cholesterol between HDL-C and remnants in plasma [13, 40]. These findings collectively suggest a strong correlation between RC levels and metabolic disorders.

Moreover, the observed correlation between RC and HUA susceptibility still exists even after controlling for various confounding factors such as BMI, age, HbA1c, SBP, DBP, indicating the potential of RC to serve as an independently HUA risk in clinical settings. In addition, it has been widely acknowledged that conventional lipid parameters contribute to the development of HUA [41], potentially leading to a misleading correlation between RC and HUA. To address this problem, a reassessment was conducted to determine whether elevated RC levels were associated with an increased HUA risk in individuals with normal routine lipid levels. These findings indicate the correlation between elevated serum RC levels and incident HUA remains robust, irrespective of the presence of hyperlipidemia.

Furthermore, whether the correlation between RC and HUA was influenced by various established risk factors was investigated through stratified analyses. This study revealed notable gender disparities in the correlation between RC and the HUA risk, with a notably stronger correlation observed in females than that in males. Interestingly, a similar trend has been observed in the correlation between RC and the risks of chronic kidney disease, diabetes, and NAFLD [36, 37, 42]. Although the exact mechanism underlying these gender-specific differences are still uncertain, sex hormones such as estrogen may play a role. Existing literature supports the influential role of estrogen signaling via Estrogen Receptor alpha (ERα) in modulating lipid and glucose metabolism [43]. Therefore, the decrease in estrogen levels following menopause may result in the dysregulation of lipid metabolism, thereby increasing the susceptibility of women to developing HUA.

Prior investigations conducted on cohorts comprising both ordinary people and individuals with coronary artery disease have suggested that RC exhibits superior predictive capabilities for the onset of hyperglycemia compared to other conventional lipid parameters [44–46], which is consistent with this study. As shown in Fig. 3, the results showed that RC had the largest AUC compared with TG, TC, HDL-C and LDL-C, indicating its superior performance in detecting HUA.

Several plausible mechanisms can be postulated to elucidate the correlation between RC and the development of HUA. First of all, the elevation of RC levels in body will lead to an induction of heightened production and utilization of free fatty acids, consequently accelerating the catabolism of adenosine triphosphate and resulting in an augmented production of serum uric acid [47]. Secondly, an elevated RC level has been found to be independently associated with a reduced estimated glomerular filtration rate and an increased risk of renal impairment, potentially leading to a diminished excretion of uric acid [36]. Finally, RC can trigger IR [48], a factor closely related to the pathogenesis of hyperuricemia. IR has been shown that it can enhance renal urate reabsorption through the stimulation of URAT1 [49] and/or the Nadependent anion co-transporter in the brush border membranes of the renal proximal tubule [49, 50].

### Study strengths and limitations

The advantage of this study lies in that the patients have been well characterized based on a large population and subgroup analyses were conducted to check whether there were differences between RC and HUA among different populations, thereby improving the reliability of the results. Nonetheless, this study is subject to certain limitations. Firstly, this study was a retrospective nature and single-center design. It is imperative that future research includes more multicenter randomized-controlled trials to investigate the correlation between RC and HUA. Secondly, it is important to note that the research population in this study was restricted to patients with T2DM. Thirdly, the measurement of RC is not currently a standard component of clinical blood lipid testing through direct means, thus only RC levels can be calculated. Fourthly, further investigation is required to elucidate the interaction correlation between RC and factors such as age, BMI, gender, hypertension, and diabetes.

## Conclusion

In conclusion, higher RC is associated with an increased HUA risk among patients with T2DM, which may be an effective indicator in identifying HUA in patients with T2DM and preventing disease progression.

### Electronic supplementary material

Below is the link to the electronic supplementary material.

Supplementary Material 1

Supplementary Material 2

Supplementary Material 3

## Data Availability

No datasets were generated or analysed during the current study.
