# Supplementary material for: Correlation between remnant cholesterol and hyperuricemia in patients with type 2 diabetes mellitus: a cross-sectional study
Source: Lipids Health Dis. 2024 May 25;23:155. doi: 10.1186/s12944-024-02148-3 (PMC11128103; doi:10.1186/s12944-024-02148-3)
Supplement: Supplementary file 1 — Supplementary Material 1 [file 12944_2024_2148_MOESM1_ESM.docx]

TableS1. Logistic regression analysis

| Variable | OR | 95%CI | *P* |
| --- | --- | --- | --- |
| Gender | 0.51 | 0.38-0.66 | <0.001 |
| Age | 0.99 | 0.98-0.99 | 0.005 |
| BMI | 1.00 | 1.00-1.01 | 0.375 |
| Waist circumference | 1.07 | 1.04-1.10 | <0.001 |
| SBP | 0.99 | 0.99-1.00 | 0.800 |
| DBP | 1.00 | 1.00-1.01 | 0.812 |
| HbA1c | 0.95 | 0.90-1.01 | 0.085 |
| ALT | 1.01 | 0.99-1.01 | 0.507 |
| GGT | 1.00 | 1.00-1.00 | 0.114 |
| Creatinine | 1.03 | 1.02-1.03 | <0.001 |
| Albumin | 1.00 | 0.99-1.01 | 0.375 |
| Drinking | 1.47 | 1.05-2.06 | 0.025 |
| DD | 1.09 | 1.00-1.19 | 0.045 |
| LLDs | 1.17 | 0.78-1.74 | 0.437 |
| Hypoglycemic drugs | 1.03 | 0.95-1.11 | 0.482 |
| RC | 1.65 | 1.42-1.92 | <0.001 |

TableS2. Association between RC and hyperuricemia in logistic regression analysis without using lipid-lowering drugs

|  | Model1 OR (95% CI)  P value | Model2 OR (95% CI)  P value | Model3 OR (95% CI)  P value |
| --- | --- | --- | --- |
| RC | 1.81 (1.59, 2.06), <0.001 | 1.82 (1.59, 2.07), <0.001 | 1.57 (1.34, 1.84), <0.001 |

Model I: None covariates were adjusted; Model II: gender and age were adjusted; Model III: BMI, waist circumference, SBP, DBP, FPG, TG, HbA1c, ALT, AST, GGT, serum creatinine, albumin, drinking, smoking, DD, LLDs, hypoglycemic drugs.
