# Supplementary material for: Correlation between remnant cholesterol and hyperuricemia in patients with type 2 diabetes mellitus: a cross-sectional study
Source: Lipids Health Dis. 2024 May 25;23:155. doi: 10.1186/s12944-024-02148-3 (PMC11128103; doi:10.1186/s12944-024-02148-3)
Supplement: Supplementary file 2 — Supplementary Material 2 [file 12944_2024_2148_MOESM2_ESM.pdf]

Correlation between Remnant Cholesterol and Hyperuricemia in Patients with Type 2 Diabetes Mellitus A Cross-sectional Study

by a03 a03

(18.07K)
Word count: 2736
Character count: 15737

Abstract

Background: The remnant cholesterol (RC) has been used as an important tool to access the metabolic syndrome (Mets) risk. However, the correlation between RC and hyperuricemia (HUA) in T2DM remains unclear. This study the to aims explore between relationship RC and HUA in T2DM with patients.

Methods: 2956 patients T2DM with to the admitted Affiliated Second Hospital and Children's Yuying Hospital of Medical Wenzhou between University 2020 and 2022 were included. The between relationship RC and HUA was evaluated with correlation Spearman's, subgroup analysis, multiple regression logistic, receiver characteristic operating (ROC) curves analysis and generalized smooth curve fitting. Total cholesterol (TC)<5.18mmol/L was defined as normal TC.

Results: RC was correlated with uric acid in T2DM with patients (Spearman's correlation =0.279, P<0.001). Multiple regression logistic analysis also showed an independent positive correlation between RC and HUA (OR=1.63, 95%CI=1.40, 1.90). In addition, a correlation non-linear between RC and HUA was identified. The under area the curve ROC (AUC) of RC (0.658, 95%CI=0.635, 0.681) was the largest compared with LDL-C, TG, HDL-C and TC. Subgroup analysis showed a more significant positive correlation among females or normal TC level groups.

Conclusion: Elevated RC is significantly and positively correlated with HUA in T2DM patients. The predictability of RC for HUA is better than that of conventional lipid indexes.

Keywords: metabolic syndrome; remnant cholesterol; hyperuricemia; diabetes; uric acid

Introduction

HUA is a abnormality metabolic syndrome caused by disturbance of purine metabolism[1]. Studies Previous have indicated that HUA is closely associated with an increased risk of syndrome metabolic, cardiac death, chronic disease kidney (CKD), disease cardiovascular (CVD) and all-cause mortality[2, 3]. Epidemiological investigations have revealed that overall the prevalence of HUA in China is currently 13.3%[4], with a notably higher occurrence in patients with diabetes mellitus (DM)[5]. The substantial rise in the HUA prevalence of poses a significant public health challenge to, with a considerable socioeconomic burden [6]. Therefore, the identification of risk factors associated with high uric acid levels in patients with DM and the discovery of potential valuable indexes can significantly enhance the and treatment management of chronic diseases.

Remnant cholesterol (RC), an innovative atherogenic lipoprotein, refers to the cholesterol content present in lipoproteins triglyceride-rich, comprising predominantly intermediate-density lipoproteins, remnants chylomicron and low-density very lipoproteins. Typically, RC is determined by subtracting the levels of LDL-C and HDL-C from the TC, as calculated from a standard lipid profile[7]. Notably, mechanistic evidence indicated that elevated concentrations of RC are associated with low-grade inflammation, which are genetically affected by insulin resistance (IR)[8-11]. A study of a subject on the epidemiology demonstrated that as the level of RC increases, there is a corresponding increase in the prevalence of T2DM, hypertension, and hypertriglyceridemia[12-15]. Furthermore, the relationship between RC and MetS is characterized by a positive feedback loop involving IR, chronic inflammation,

hypertension and abnormal lipid metabolism[16-19]. RC has the impact on these factors and its reciprocal relationship with the results in the accelerated progression of MetS[16, 17].

Previous studies have explored the correlation between conventional lipid parameters such as TC or TG and HUA[20-23]. In addition, Wang et al. found a positive correlation between elevated RC and HUA in American adults[24]. However, the precise correlation between RC and HUA in T2DM patients remains unclear. Consequently, this study to aims investigate the potential link between RC and HUA in patients with T2DM cross-sectional through a analysis, so as to determine the viability of RC as a novel and practical biomarker for the diagnosis of HUA.

Methods

Subjects and study design

The study's inclusion criteria consisted of a diagnosis for T2DM on the criteria established by the World Health Organization, a minimum age of 20 years, biochemical complete parameters, and information clinical. Exclusion were as criteria follows: (1) Patients with a history of using diuretics or other medications potentially impacting uric acid metabolism over the past two months; (2) Patients with acute inflammatory or infection disease; (3) Patients with acute diabetic complications such as ketoacidosis or hyperosmolar state (coma); (4) Patients with chronic kidney disease accompanied by eGFR less than 60 mL/min; (5) Patients with severe chronic illness, such as cardiovascular diseases and cancer

Anthropometric measurements

The following collected were data admission at: Duration of diabetes (DD), hypertension history of, hypoglycemic drugs, smoking habits, drugs lipid-lowering (LLDs), intake alcohol, and measurements physical, including waist circumference height, weight and blood pressure. Specifically, the alcohol status definitions of, BMI, hypertension and smoking were previous studies described in [25].

To obtain blood samples, 4-5 mL blood venous was collected the morning next after patients fasted overnight for 12 h. LDL-C, ALT, TC, serum uric acid, glycosylated hemoglobin (HbA1c), TG, HDL-C, aspartate aminotransaminase (AST), albumin, creatinine, gamma-glutamyl transpeptidase (GGT) and FPG were determined as previously described [12]. Blood lipids were measured using enzymatic method and Olympus automatic biochemical analyzer.

RC was calculated as the formula following: RC=TC—HDL-C—LDL-C. RC values were into four groups divided based on quartiles (Q1–Q4). HUA was defined as a uric acid level exceeding 420μmol/L in males and 360μmol/L in females[4]. The classification of HDL-C, TC, LDL-C and TG was determined in accordance with the on the Guidelines treatment and prevention of blood lipid abnormalities in Chinese adults[26]. The TG level of was defined as TG normal and hypertriglyceridemia (cutoff value=1.70mmol/L). The TC level of was defined as normal TC and hypercholesterolemia (cutoff value=5.18mmol/L). The HDL-C level of was defined as normal HDL-C and low HDL-C (cutoff value=1.04 mmol/L). The level of LDL-C was defined as normal LDL-C and high LDL-C (cutoff value=3.37 mmol/L).

Statistical analysis

The relationship between RC and the presence of HUA was assessed with Binary logistic regression models. In Model 1, no adjusted covariate was. In Model 2, there were adjustment for gender and age. Based on Model 2, BMI, waist circumference, SBP, DBP, HbA1c, ALT, GGT, serum creatinine, albumin, drinking, smoking, DD, LLDs, hypoglycemic drugs were added to Model 3 as covariates. Mediation was performed analysis using the mediation parallel model, with indicators individual serving mediators as. The potential gender, impacts of BMI, hypertension, TG, HDL-C, age, TC, as well as LDL-C on the relationship between RC and HUA were examined through subgroup analyses. To identify the potential nonlinear relationship between RC and HUA probabilities, smooth curve fitting is used. Evaluate diagnostic effect the of RC on HUA through research. Finally, to avoid the impact ROC curve of LLDs on the correlation between RC and HUA, we also a conducted analysis sensitivity (n=2185) on who did not participants use LLDs. Using top filling statistical software and R for statistical analysis, P<0.05 is considered significant.

Results

Baseline characteristics

A total of 2956 participants aged from 25 to 90 years old included were in this study, with the prevalence of HUA of 27.8%. The characteristics population of the participants based on serum RC quartiles (Q1: <0.34; Q2: 0.34–0.53; Q3: 0.53-0.80; Q4: >0.80) are presented in Table 1. Compared bottom quartile with the, the prevalence of HUA and hypertension was higher in those in the top quartile of RC, with elevated levels of body waist circumference, weight, diastolic and systolic blood pressure, FPG,

creatinine, uric acid, TC, and TG. In contrast, the HDL-C levels were lower ($P<0.001$) (Table1).

Correlation between metabolic parameters and RC

The correlation between RC and metabolic parameters, as measured by Spearman's correlation coefficient, can be found in Table 2. It is evident that RC is positively correlated with BMI, WC, SBP, DBP, FPG, TC, TG, uric acid, and negatively correlated with HDL-C, LDL-C (Figure 1 and Table 2).

Relationship between RC and HUA risk

Three logistic regression multivariate were developed to examine the relationship between HUA and RC (Table 3 and Table S1). In the model unadjusted, RC was correlated positively with HUA probabilities [OR = 1.92, 95% CI: (1.69, 2.17)]. The relationship still existed in the Model 2 [OR = 1.92, 95% CI: (1.69, 2.17)] and Model 3 [OR=1.63, 95% CI: (1.40, 1.90)]. Moreover, with the compared lowest level of RC (Q1) in Model 3 (P for trend < 0.001), the HUA risk of the subjects in quartiles 3 and 4 increased by 0.71 and 1.36, respectively.

Subgroup analysis to relationship assess the between RC and HUA

A comprehensive analysis subgroup was performed to evaluate the consistency of the correlation between RC and HUA risk across different demographic contexts. As shown in Table 4, the between relationship RC and HUA risk was among stronger female and normal TC participants than in males and hypercholesterolemia participants (P interaction<0.05). In all participants, a correlation was positive observed in the non-linear correlation, with inflection points of 7.443 (figure2). In addition, figure

3 displays the smooth curves showing the positive correlation between RC level and HUA in most groups.

1

ROC analysis

Figure 4 shows the ability of ROC curve of RC, HDL-C, TC, TC, LDL-C and TG in identifying HUA risk. The AUC for RC was significantly greater than TC, TG, HDL-C and LDL-C through the ROC analysis (0.658, 95%CI=0.635, 0.681), with sensitivity of the 60.8%, the specificity of 63.3% cutoff of and the 0.54 (Table 5).

Sensitivity analysis

Considering the effect probable of using LLDs on the between association RC and HUA, performed we a analysis sensitivity to this association determine after the participants excluding who the took LLDs (n = 771). In the multivariable-adjusted logistic model, RC was positively correlated with HUA probabilities (Supplementary Table 2).

Discussion

The extensive studies have revealed a positive correlation between RC and an increased uric acid, and the risk of HUA among patients with T2DM. Moreover, the results of subgroup analyses indicated a robust positive relationship, particularly in females and patients with normal TC levels. Furthermore, findings reveal the a non-linear relationship between RC and the risk of HUA. In addition, RC has the superior predictive ability for HUA compared with conventional lipid parameters.

In view of its rising substantial clinical disorders, HUA has become a significant public health issue[2, 27-29]. Cao et al. conducted a large-scale prospective cohort study involving 58,5 Chinese individuals. The study revealed an incidence of HUA of 12.1%

with a median follow-up period of 2.5 years. In a separate prospective cohort study conducted in China, Zhang et al. observed an occurrence of HUA in 28% of patients over 6 years[30]. Notably, the Risk Atherosclerosis in Communities Study including 9451 Americans regularly consumed fructose - high corn syrup, such as sweetened soda sugar, reported a 34.8% development rate of HUA during a 6-year follow-up[31]. In this study on patients with T2DM (58.9 ± 15.2 years, 58.8% males), it observed 27.4% of T2DM adults with HUA. With the rapid changes in Chinese habits dietary and the rapid Westernization lifestyle of, the incidence of hyperuricemia in the population Chinese will continue to rise, leading to adverse health serious consequences. Therefore, studying the risk factors of hyperuricemia can help prevent and treat cardiovascular diseases in the early stages.

The impact of dyslipidemia on the HUA development has been investigated in various epidemiological and clinical studies. NHANES III indicated a significant correlation between TG and TC levels and UA levels in the serum of ordinary adults[22]. A retrospective population-based study involving 3884 medical examined patients collected from Gansu, China, revealed a positive correlation between elevated TG levels and HUA[21]. Recent studies has shown that abundant RC in triglyceride (TG) lipoprotein, such as intermediate-density lipoprotein, chylomicron remnants, and very-low-density lipoprotein [32], can contribute to various atherosclerotic effects, including the upregulation of proinflammatory cytokines, activation of monocytes, and increased production of thrombogenic factors [11, 32]. Adverse cardiovascular events associated with RC have been documented in numerous clinical studies. However, RC has been proposed as a potential means of identifying individuals at higher risk for T2DM,

cardiovascular diseases, chronic kidney disease, fatty liver and Mets[33-38], no studies have reported on the relationship between the prevalence of HUA and increased RC yet.

This study showed that RC was positively correlated with TG, DBP, BMI, FPG, WC, SBP and negatively correlated with HDL-C, which is consistent with previous studies. Additionally, it was found that the correlation between RC and TG was the strongest compared to that with RC and that with other components of MetS. This finding is consistent with previous studies, suggesting that TG is primarily transported by remnants and that the concentration of TG significantly increases with elevated levels of RC[14, 39]. In addition, it was observed that as RC levels increased, HDL-C levels decreased due to the exchange of triglycerides and cholesterol between HDL-C and remnants in plasma[13, 40]. These findings collectively suggest a strong correlation between RC levels and metabolic disorders.

Moreover, the observed correlation between RC and the susceptibility to HUA persisted even after controlling for various confounding factors such as BMI, age, HbA1c, SBP, DBP, indicating the potential of RC to serve as an independently risk of HUA in clinical settings. In addition, it has been widely acknowledged that conventional lipid parameters contribute to the development of HUA[41], potentially leading to a misleading correlation between RC and HUA. In order to issue this problem, a reassessment was conducted to determine whether elevated RC levels were associated with an increased risk of HUA in individuals with normal routine lipid levels. The findings indicate the relationship between elevated serum RC levels and incident HUA remains robust, irrespective of the presence of hyperlipidemia.

Furthermore, whether the correlation between RC and HUA was influenced by various established risk factors was investigated through stratified analyses. This study revealed notable gender disparities in the correlation between RC and the risk of HUA, with a notably stronger correlation observed in females than that in males.
Interestingly, a similar trend has been observed in the relationship between RC and the risks of chronic kidney disease, diabetes, and NAFLD[36, 37, 42]. Although the exact mechanism underlying these gender-specific differences are still uncertain, sex hormones such as estrogen may play a role. Existing literature supports the influential role of estrogen signaling via Estrogen Receptor alpha (ERα) in modulating lipid and glucose metabolism[43]. Therefore, the decrease in estrogen levels following menopause may result in the dysregulation of lipid metabolism, thereby increasing the susceptibility of women to developing HUA.

Prior investigations conducted on cohorts comprising both ordinary people and individuals with coronary artery disease have suggested that RC exhibits superior predictive capabilities for the onset of hyperglycemia compared to other conventional lipid parameters[44-46], which is consistent with this study. As Figure 3 shown in, the results showed that RC largest AUC had the with compared TG, TC, HDL-C and LDL-C, superior performance indicating it in detecting HUA.

Several plausible mechanisms can be postulated to elucidate the correlation between RC and the development of HUA. First of all, the elevation of RC levels in body will lead to an induction of heightened production and utilization of free fatty acids, consequently accelerating the catabolism of adenosine triphosphate and resulting in an augmented production of serum uric acid[47]. Secondly, an elevated RC level has

been found to be independently associated with a reduced eGFR and an increased risk of renal impairment, potentially leading to a diminished excretion of uric acid[36]. Lastly, RC can trigger IR[48], and IR is a factor closely related to the pathogenesis of hyperuricemia. IR has been shown that it can renal urate enhance reabsorption through the URAT1 stimulation of [49] and/or the Nadependent anion brush border co-transporter in the membranes of the proximal tubule renal [49, 50].

Study strengths and limitations

The advantage this study of lies in that we have characterized well the subjects based large population on a and conducted subgroup analysis to check whether there were differences between RC and HUA among different populations, thereby improving the results reliability of the. Nonetheless, this study is subject to certain limitations. Firstly, this study was a retrospective nature and single-center design. It is imperative that future research includes more multicenter randomized-controlled trials to investigate the correlation between RC and HUA. Secondly, it important to is note that the population research in this study was restricted to patients with T2DM. Thirdly, the measurement of RC is not currently a standard component of clinical blood lipid testing through direct means, thus only RC levels can be calculated. Fourthly, further investigation is required to elucidate the interaction relationship between RC and such as factors age, diabetes, BMI, gender, hypertension, and.

Conclusion

In conclusion, higher RC levels are associated increased risk with an of HUA among patients T2DM with. It may be an effective indicator to help identify HUA in T2DM patients and prevent disease progression.

Correlation between Remnant Cholesterol and Hyperuricemia in Patients with Type 2 Diabetes Mellitus A Cross-sectional Study

ORIGINALITY REPORT

8%	1%	7%	1%
SIMILARITY INDEX	INTERNET SOURCES	PUBLICATIONS	STUDENT PAPERS

PRIMARY SOURCES

1	Shouxing Yang, Jing Xu. "Elevated small dense low-density lipoprotein cholesterol to high-density lipoprotein cholesterol ratio is associated with an increased risk of metabolic dysfunction associated fatty liver disease in Chinese patients with type 2 diabetes mellitus", Journal of Diabetes Investigation, 2024<br>Publication	4%
2	Wei Zhou, Nianchun Shan, Jie Wei, Yang Zhou, Meichao Men. "Cross-sectional and longitudinal associations between lipid accumulation product and hyperuricemia", Nutrition, Metabolism and Cardiovascular Diseases, 2022<br>Publication	1%
3	Jae-Sung Nam, Yun-Ho Roh, Jungghi Kim, Suk-Won Chang et al. "Association between diabetes mellitus and chronic rhinosinusitis	1%

with nasal polyps: A population-based cross-sectional study", Clinical Otolaryngology, 2021
Publication

4	bmcneurol.biomedcentral.com<br>Internet Source	<1%
5	"BASL abstracts", Gut, 5/1/2003<br>Publication	<1%
6	Liang, Fan. "Olive Oil and Nuts Among Two Ethnic Groups: An Evaluation of Their Acceptability, a Survey and Systematic Review of Previous Interventional Evidence of Their Effects on Cardiovascular Health, and Results from an RCT Intervention Study", University of Northumbria at Newcastle (United Kingdom), 2022<br>Publication	<1%
7	Submitted to University of Glasgow<br>Student Paper	<1%
8	www.ncbi.nlm.nih.gov<br>Internet Source	<1%
9	Md Azharuddin, Prem Kapur, Ritu Mishra, Shakir Saleem, Ashok Kumar Gupta, Mohammad Adil, Manju Sharma. "Predictor for cardiovascular risk in patients with type-2 diabetes mellitus", Clinical Epidemiology and Global Health, 2021<br>Publication	<1%

10	da Costa, Cibelle Neiva Cavalcanti Mariano. "Biochemical and Molecular Characterisation of The Dyslipidaemia in Portugal", Universidade de Lisboa (Portugal), 2021<br>Publication	<1 %

Exclude quotes	Off	Exclude matches	Off
Exclude bibliography	Off		
